# Comparative transcriptomic analysis of bovine papillomatosis

**DOI:** 10.1186/s12864-018-5361-y

**Published:** 2018-12-19

**Authors:** Débora M. Barreto, Gerlane S. Barros, Lucas A. B. O. Santos, Rosilene C. Soares, Marcus V. A. Batista

**Affiliations:** 1 Laboratory of Molecular Genetics and Biotechnology, Department of Biology, Center for Biological and Health Sciences, Federal University of Sergipe, São Cristóvão, Sergipe Brazil; 2 Department of Morphology, Federal University of Sergipe, São Cristóvão, Sergipe Brazil

**Keywords:** Bovine papillomavirus, RNA-seq, Differently expressed genes, Keratinocyte, Apoptosis, Immune response

## Abstract

**Background:**

Bovine papillomavirus (BPV) belongs to the Papillomaviridae family and infects epithelial cells of bovines and closely related animals, causing hyperproliferative lesions known as warts or papillomas, which may regress or progress to form benign or malignant tumors. The virus enters the host cell and interacts with it by altering the regulation of genes that are responsible for controlling the cell cycle, thus triggering lesion formation. It is not yet known which host genes are regulated by viral infection. Therefore, the objective of this study was to make use of next-generation RNA sequencing methods to identify differentially expressed genes associated with BPV infection, which might elucidate possible marker genes that could be used to control the disease.

**Results:**

Transcriptome analysis revealed that 1343 genes were differentially regulated (FDR < 0.05). A comparison of gene expression in infected and noninfected cows indicated that 655 genes were significantly upregulated, and 688 genes were significantly downregulated. Most differentially expressed genes were associated with BPV infection pathways, which supports the hypothesis that viral infection was the mechanism associated with this regulation.

**Conclusions:**

This is the first study that focused on a large-scale evaluation of gene expression associated with BPV infection, which is important to identify possible metabolic pathways regulated by host genes for lesion development. In addition, novel targets could be identified in order to find ligands that interact with BPV, with the aim of interrupting the infection cycle.

**Electronic supplementary material:**

The online version of this article (10.1186/s12864-018-5361-y) contains supplementary material, which is available to authorized users.

## Background

With a herd of 218.23 million head of cattle and annual milk production of 33.62 billion liters [1], Brazil has the second largest cattle herd in the world and is the fifth largest producer of milk [2, 3]. US Department of Agriculture (USDA) data show that Brazil is the second largest producer of commercial beef of the world, with annual production of 2.586 million tons. These data show the strategic position of cattle in the country’s economy. However, some pathogens are known to affect the level of this production by causing severe damage to animals and great losses for producers. Among these pathogens is the bovine papillomavirus (BPV), which causes bovine papillomatosis in cattle in Brazil and several other countries. The virus mainly affects young animals, causing cutaneous and mucosal lesions that can be minimized using chemicals, surgical removal or cauterization of lesions, and autogenous vaccine usage. If left untreated, lesions may develop into malignant tumors [4, 5].

The process of viral infection is coupled to replication of the viral genome. This process depends on different host genetic factors. During infection, host genes are inhibited or activated by pathogen proteins [6]. Studies have shown that the virus regulates these genes to ensure an increase in the copy number of the viral genome and thereby trigger the infection process [6, 7]. However, it is not yet known which of these host genes are regulated by viral infection.

In the present study, we have analyzed the differential expression of genes involved in the host-pathogen relationship, thus contributing to the development of new markers associated with viral infection. Next-generation RNA sequencing technology (RNA-seq) allows detailed profiling of gene expression levels. In this type of sequencing, a large amount of RNA sequence information is generated and requires automated analysis. This approach has been used successfully to study bovine mammary tissue [8–15] and to analyze differentiated keratinocytes during human papillomavirus infection [6], but none of these studies considered the effect of gene expression during bovine papillomavirus infection on the transcriptome. In this study, we used RNA-seq to gain unprecedented insight into the regulatory mechanisms underlying the effect of gene expression during bovine papillomavirus infection.

## Results

**Fig. 1:**
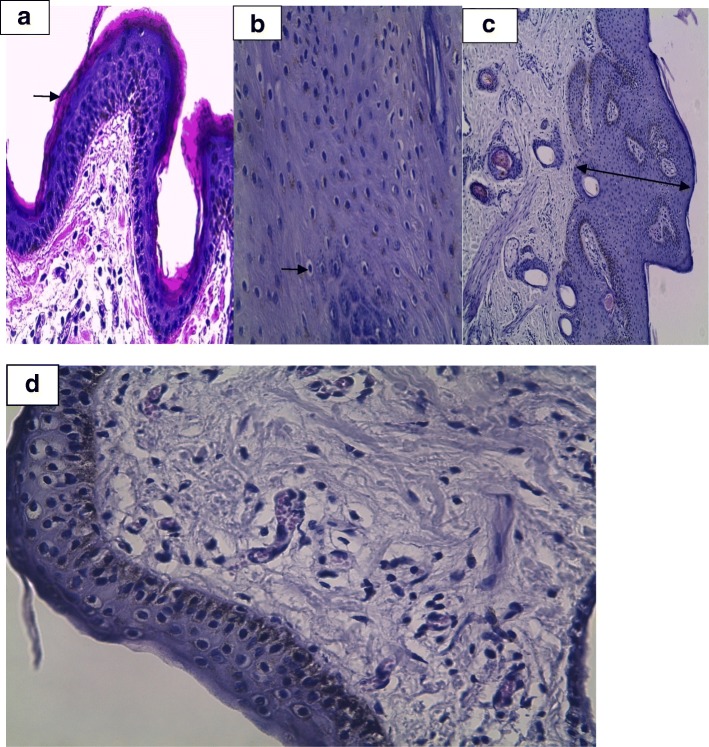
Photomicrographs of a wart stained by hematoxylin and eosin. **a** fragment of a papilloma. Hyperkeratosis of the epithelial layer with irregularity of the epidermis. Magnification: 40×. **b** visualization of koilocytosis at 40× magnification. **c** acanthose at 10× magnification. **d** noninfected animal tissue evidencing the epithelial layer without the presence of hyperkeratosis and acanthosis

Histopathological analysis confirmed the presence of papillomatous lesions in three samples. The presence of hyperkeratosis of the epithelial layer, koilocytosis and acanthosis (Fig. 1a, b, c and d) were visualized through photomicrography. Such changes are characteristic of BPV infection [12]. In healthy animals, there was no histology compatible with papillomatous lesions. In addition, PCR detected the presence of the virus in all papillomatosis samples, and samples from healthy animals tested negative for viral DNA.

### Sequencing, transcript mapping, and gene expression analysis

Six libraries generated with RNA samples from cows with papillomatous infection and healthy animals were sequenced. RNA-sequencing generated a total of 121,722,238 raw paired reads with an average of 20 M reads per library. The alignment of reads showed that 87.56% (93,261,478) mapped to the bovine genome (UMD3.1.77, which has 24,617 annotated genes) (Additional file 1). Of the mapped reads, 82.38% mapped to unique positions, 14.15% mapped to multiple positions, and 3.62% were discordant alignments.

The distribution of transcript expression is shown in Additional file 2. A total of 14,213 genes were expressed, of which 1343 were significantly differentially regulated (FDR < 0.05) in a comparison of gene expression between infected cows and healthy animals. Of these, 655 genes were significantly upregulated and 688 were significantly downregulated in infected animals (Additional file 3).

### qPCR validation of RNA-sequencing results

Our qPCR results confirm the differential expression patterns of RNA-seq obtained for infected and healthy animals. Differential gene expression identified by using qPCR was statistically significant (*P* < 0.0003). Differences in gene expression between the case and control groups were statistically significant when both methods were used, RNA-seq and qPCR (Table 1).

**Table 1:** Results of qPCR validation of RNA-sequencing data

Gene	qPCR		RNA-Seq	
	Fold change infected vs non-infected	*P*-value	Fold change infected vs non-infected	FDR *P*-value
BOLA	0.16	0.0003	0.12	0.0010
CST6	0.11	0.0001	0.17	0.0010
KRTAP3–1	0.02	0.0001	0.09	0.0010
KRT78	6.27	0.0001	11.81	0.0010
UBA7	1.22	0.0001	2.37	0.0010
BNIP3	7.46	0.0001	17.11	0.0057
IVL	3.27	0.0001	9.33	0.0010
DAPL1	0.04	0.0001	0.13	0.0010
FABP4	26.35	0.0001	34.87	0.0010

### Biological function enrichment and pathway analysis of differentially expressed genes

Of the differentially expressed genes, we identified 122 genes with immune function, 489 related to the cell cycle, 112 involved in the process of ubiquitination, and 42 in the process of apoptosis. The top 24 differentially expressed genes in infected animals and their functions are listed in Table 2. We also found 26 genes involved in the process of keratinization and 162 in the process of gene expression (Additional file 4).

**Table 2:** Twenty-four top differentially expressed genes and their functions

Gene symbol	Gene name	Fold change	FDR	Gene function
CXCL8 IL8	C-X-C motif chemokine ligand 8	582.4973	0.0010	Participates in the immune response, cellular response to tumor necrosis factor, regulation of cell proliferation and cell cycle arrest
ISG15	ISG15 ubiquitin-like modifier	20.6836	0.0010	Plays a key role in the innate immune response to viral infection, ubiquitination and apoptosis
IL33	Interleukin 33	0.4434	0.0246	Participates in the response to viral infection, regulation of cell proliferation and RNA transcription, ubiquitination and apoptosis
FABP4	Fatty acid binding protein 4	34.8777	0.0010	Involved in cell cycle, cell differentiation, positive regulation of cell proliferation and positive regulation of inflammatory response
PLAUR	Plasminogen activator, urokinase receptor	8.9016	0.0010	Plays a role in positive regulation of epidermal growth factor and involved in the apoptosis
HMOX1	Heme oxygenase 1	5.5229	0.0019	Positive regulation of apoptotic process, regulation of transcription and cell proliferation
CTNNBIP1	Catenin beta interacting protein 1	3.9524	0.0010	Involved in cell cycle, cell proliferation, regulation of transcription and regulation of inflammatory response
CXCL2	chemokine (C-X-C motif) ligand 2	3.3373	0.0010	Plays role in the immune response and cell proliferation
YOD1	YOD1 deubiquitinase	5.2337	0.0010	Plays a role in the ubiquitin pathway
RNF19B	Ring finger protein 19B	2.8067	0.0010	Plays a role in the ubiquitin pathway
UBA7	Ubiquitin like modifier activating enzyme 7	2.3716	0.0010	Plays a role in the ubiquitin pathway
S100A9	S100 calcium binding protein A9	39.4951	0.0034	Involved in the immune response, regulation of inflammatory response and apoptosis
S100A8	S100 calcium binding protein A8	10.9851	0.0010	Involved in the immune response, regulation of inflammatory response and apoptosis
CASP14	Caspase 14	53.4185	0.0010	Plays a role in the apoptosis
BNIP3	BCL2 interacting protein 3	17.1102	0.0057	Participates in defense response to virus, and negative regulation of apoptotic process
TMEM79	Transmembrane protein 79	2.5117	0.0034	Structural molecule activity
TERT	Telomerase reverse transcriptase	4.4637	0.0143	Involved in cell cycle, regulation of transcription and structural molecule activity
HEYL	Hes related family bHLH transcription factor with YRPW motif-like	0.2704	0.0010	Involved in cell differentiation
JUNB	JunB proto-oncogene, AP-1 transcription factor subunit	2.8321	0.0063	Participates in cell proliferation, cell cycle and regulation of transcription
CSRP2	Cysteine and glycine rich protein 2	5.8161	0.0010	Involved in cell differentiation
CTNNBIP1	Catenin beta interacting protein 1	3.9524	0.0010	Cell proliferation and differentiation, involved in acute inflammatory response and regulation of transcription
NME2	non-metastatic cells 2, protein (NM23B) expressed in	2.2077	0.0197	Regulation of apoptotic process and transcription
ALOX12B	Arachidonate 12-lipoxygenase, 12R type	3.9381	0.0010	Regulation of transcription
CHP2	Calcineurin like EF-hand protein 2	2.7195	0.0019	Participates of cell proliferation and regulation of transcription

**Fig. 2:**
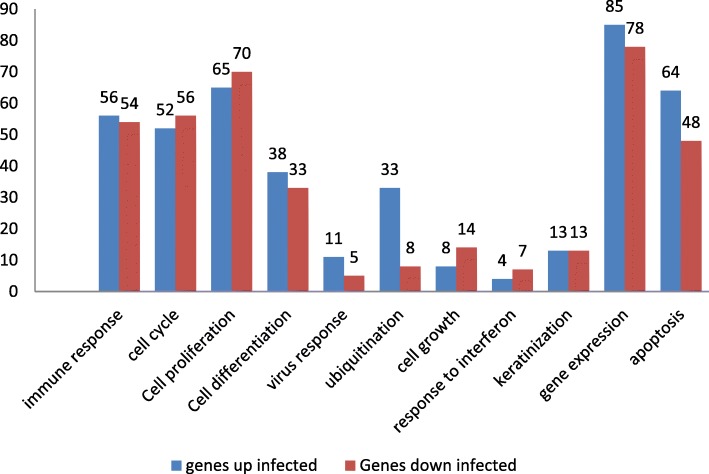
Differentially expressed genes in infected and healthy animals that are significantly enriched in several molecular and cellular functions

Figure 2 shows the genes that were upregulated and downregulated in infected animals and their molecular functions. Particularly, among these GO functional classes, the genes associated with ubiquitination and apoptosis were expressed at higher levels in infected animals, suggesting that the corresponding genes might be related to the pathogenic mechanism of BPV.

**Fig. 3:**
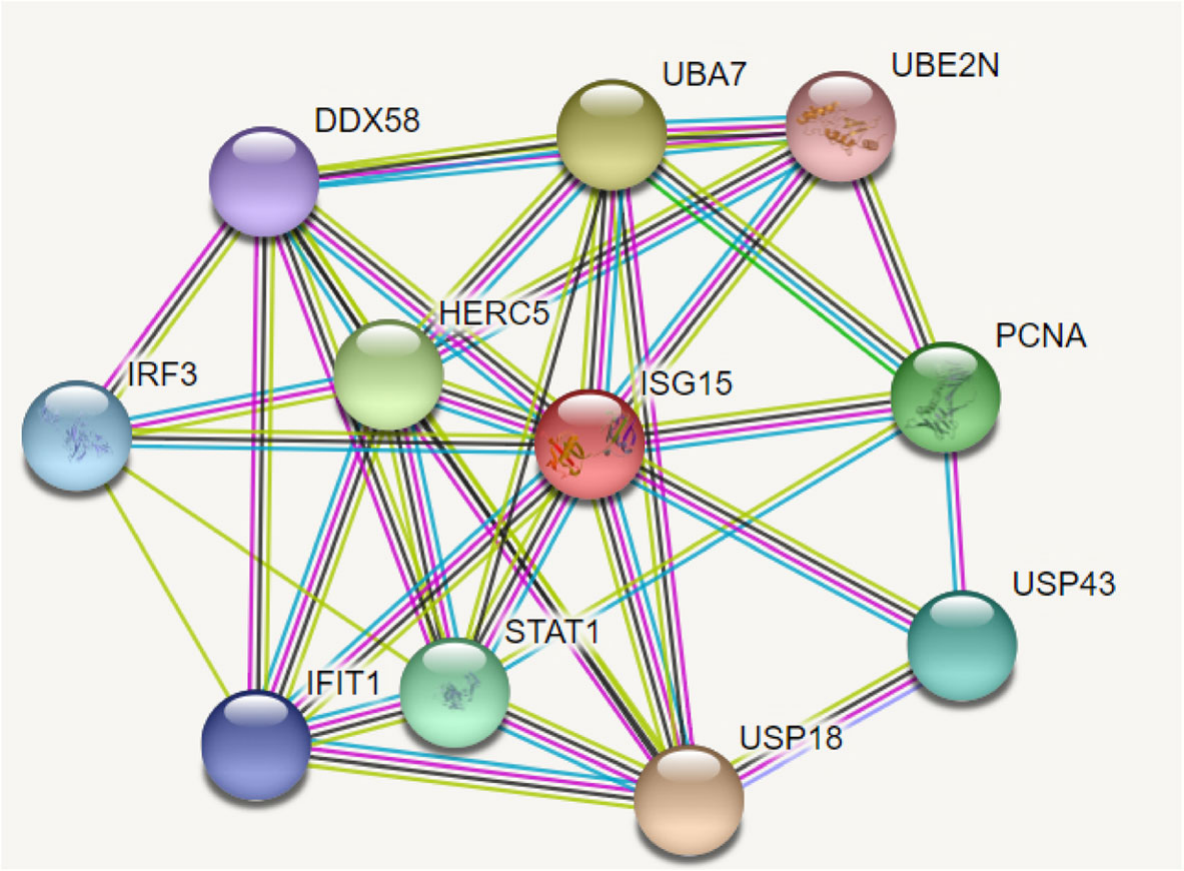
Interaction between the upregulated differentially expressed gene ISG15 and its functional cellular partners. Colored nodes represent the query protein and first shell of interactors. Filled nodes are related to known or predicted 3D structures. Empty nodes correspond to proteins of unknown 3D structure. Colored lines represent known, predicted, and other interactions. The light blue line color is associated with known interactions from curated databases; the pink line represents the experimentally determined interactions; the green line represents interactions predicted from gene clusters; the red line is associated with predicted interactions according to gene fusions; the dark blue line represents predicted interactions of gene co-occurrence; yellow, black and gray lines represent other interactions

KEGG analysis identified several metabolic pathways associated with papillomavirus infection. The most commonly encountered pathways were cell adhesion of molecules, processing and presentation of antigen, differentiation of TH1 and TH2 cells, and the pathway of human papillomavirus infection (Additional file 4). In those metabolic pathways, protein-protein interactions were analyzed, and the associations between proteins and shared functions was possible to verify. For example, Fig. 3 shows the predicted and known interactions of ISG15 protein, which participates in the metabolic pathway of human papillomavirus infection. This gene plays a key role in the innate immune response to viral infection and ubiquitination. The ISG15 cellular partners were analyzed, and among the many functions, all had in common the functions of cellular response to viruses and ubiquitination. Two of the ISG15 partners were differentially expressed in this study, UBA7 and HERC5, which were upregulated in infected cows, suggesting that they might be related to viral pathogenicity.

## Discussion

In this study, we identified that BPV infection in the animal modified the expression profile of some of the host genes that were upregulated in infected animals, such as the keratin genes TMEM79, IVL, FOXN1, and SOSTDC1. A previous study [6] presented a change in the expression profile of keratinocytes in HPV-infected cells. The high expression of these cells is directly influenced by the pathogen, as the virus infects these cells and uses its machinery to replicate and transcribe viral genes. In another study [13], high expression of the TREM1 gene was verified on the cell surface of epithelial tissue infected by *Leishmania*. In this study, the overexpression of TREM-family genes was also verified in virus-damaged tissues. In this way, it is possible to infer that the gene in question is being expressed via activation of immune system cells by the host as a response to infection.

Some genes related to the immune process, specifically the response to viruses that activate the MHCI pathway, were found: 28 were upregulated genes, and only 8 were downregulated (STMN1, KYNU, CCL26, CCL14, CXCL14, HLA-A, CCL14, and IL1F10). In this study, we have identified 120 genes with a role in regulating the inflammatory process and immune response, for example, the S100A8 and S100A9 genes were upregulated in infected animals along with their partners S100A12, IL8 and IL1B genes. This expression profile was also observed in a previous study [14], when S100A8 and S100A9 protein expression in the skin of epidermodysplasia verruciformis (EV) patients was investigated. In non-lesioned skin, both proteins were occasionally detectable in the keratinocytes of the granular layer or were completely lacking. The exact mechanisms underlying the regulation of these proteins remains to be determined; however, it can be speculated that the S100A8/A9 genes might exert their effects during carcinogenesis.

Of the genes that participate in the immune response, 61 were found to be downregulated in infected animals, such as the CCL20 gene, which is responsible for chemotaxis in dendritic cells (DC), effector/memory T-cells, and B-cells and plays an important role on skin and mucosal surfaces under homeostatic and inflammatory conditions, as well as in pathogenesis, including that of cancer and autoimmune diseases.

Sperling et al. [15] proposed a novel molecular mechanism for how HPV8 might disrupt innate immunity in the skin. They found reduced numbers of antigen-presenting Langerhans cells in epidermodysplasia verruciformis lesions and showed the reduction to be a consequence of HPV8-mediated suppression of CCL20. They showed that CCL20 is expressed in the uppermost differentiated layers of normal skin epidermis but is almost absent in HPV8-positive lesioned skin of EV patients. The virus might use this form to escape the host immune response.

Genes involved in cell proliferation and growth were also found to be differentially expressed. These genes include members of the SOX family of transcription factors mainly involved in the regulation of the malignant process through their ability to regulate numerous cancer markers, including those of cell proliferation, apoptosis, survival, invasion, migration, and differentiation [16]. Studies showed that SOX14 overexpression decreases viability and promotes apoptosis by altering the expression of apoptosis genes, promoting accumulation of p53 [16, 17]. In this study, the QSOX gene was found to be a member of the SOX family and possibly involved in the regulation of apoptosis. The presented results indicate that QSOX, as well as the SOX14 gene, may be associated with the process of apoptosis. These viruses have developed numerous strategies in order to block host-mediated apoptosis, contributing to cancer development. Programmed cell death, known as apoptosis, is a pivotal mechanism of cell death that plays an important role during diverse biological processes, including development, cell differentiation, and proliferation [18].

Several mechanisms have been found to cause bcl-2 deregulation and cancer, including activation of chromosomal translocation and upregulation upon viral infection, for instance, upregulated bcl-2 expression has been described in different tumors, including premalignant and malignant lesions of the uterine cervix induced by human papillomaviruses [19]. p53 has a significant role in cell cycle control; therefore, the loss of its suppressive function has been reported in different types of human neoplasia and animal tumors, such as BPV-induced tumors [20]. However, studies concerning the expression and impact of the anti-apoptotic protein bcl-2 and the tumor suppressor p53 in BPV-induced cutaneous tumors are lacking. In the study of Bocaneti [21], altered bcl-2 and p53 immunoreactivity in bovine cutaneous fibropapillomas was observed, suggesting the involvement of these two proteins in cutaneous neoplastic transformation through an impaired apoptotic process.

The E5, E6, E7, and E2 proteins of PVs are known to control the expression of host genes. E6 and E7 control the keratinocyte cell cycle, cell differentiation, and apoptosis programs. Thus, the alterations in gene expression that we observed in this study might be attributed to the functions of BPV oncoproteins. These changes are important for the infection cycle of the virus and persistent infection [9, 10]. These oncogenes interact with several different host proteins and thus could affect their expression. Therefore, in this study, we have identified several genes that possibly participate in papillomavirus infection pathways, which makes an important contribution to existing knowledge on the papillomavirus infection cycle, providing novel possible markers that could be used for the development of new prognostic and treatment methods.

## Conclusions

In conclusion, we report RNA-Seq analysis of changes in the cattle transcriptome caused by BPV infection. Infection caused massive changes in the expression profile of keratinocyte, immune system, cell proliferation, and apoptosis genes. These changes mainly resulted in an expression profile characteristic of viral infection rather than tumor progression. The large dataset we have developed opens up the possibility of a deeper understanding of the cycle of BPV infection in the host. Therefore, this study serves as the basis for the realization of new studies aimed at understanding the functions of genes that could act in specific pathways during viral infection, increasing our understanding regarding the infectious cycle in the animal. In addition, this study provides information that could be used to find molecular targets for possible drugs that interrupt the cycle of infection of BPV, thus preventing papillomatosis.

## Methods

### Animals, management, and sampling

The animals used in this study were owned by the São José farm in the Central Agreste region of the state of Sergipe, northeastern Brazil. Sample collection was performed by qualified veterinary medical professionals and registered in the class council, according to the procedures established by the corresponding ethics committee. Briefly, six female Girolando dairy breeders in the Central Agreste region of the state of Sergipe, northeastern Brazil, were used. Animal care, management, and usage procedures were approved by the ethics committee on production animals of the Federal University of Sergipe (protocol number: 05/14). Three animals with papillomatosis and three healthy animals were used. We collected samples of skin from the neck region of each animal. We used different animals because recent studies have detected the DNA of HPV in various parts of the host, such as peripheral blood mononuclear cells (PBMC), plasma, urine, semen, spermatozoa, trophoblasts, and the bladder. These studies have shown that PVs are not strictly epitheliotropic [22–28]. In addition, each lesion was analyzed in triplicate. All animals were females approximately 2 years old. Each sample was divided into three parts: one was used to confirm the presence of BPV in the lesion and was then wrapped in foil and stored at − 20 °C; another, for confirmation of the injured tissue, was placed in 10% formalin; and another, for the evaluation of gene expression, was placed in RNAlater and then stored at − 80 °C.

### Histopathological analysis

Formalin-fixed tissue samples were processed by standard paraffin wax techniques. Samples were then cut into 3–5-μm sections and stained by the hematoxylin and eosin method (HE). Slides were evaluated microscopically at increasing magnifications (5×, 10×, 20×, 40×).

### Viral detection

Each sample was individually macerated for total DNA extraction with the aid of the DNeasy Blood and Tissue kit (Qiagen), following the supplier’s instructions. About 100 ng of DNA were used for polymerase chain reaction (PCR) using the general detection primers FAP59/64 [29]. All amplification reactions were prepared to a final volume of 25 μL using the Master Mix PCR kit (Promega), according to the supplier’s protocol. All primers used were diluted to 10 μM.

### RNA isolation and sequencing

Total RNA (~ 15 mg/sample) were purified using the Realipred™ RNA Tissue Miniprep System Kit (Promega) according to the manufacturer’s instructions. The concentration of RNA was measured with Nanodrop ND-2000 instrument (NanoDrop Thermo Scientific, Wilmington, DE, USA), and the quality was assessed with an Agilent 2100 Bioanalyzer (Agilent Technologies, Santa Clara, CA, USA). Libraries were generated from 1000 ng of total RNA using the TruSeq stranded mRNA Sample Preparation LS Protocol Kit (Illumina Inc. San Diego, CA, USA) as per the manufacturer’s recommendations. Libraries were quantified using the Kapa Library Quantification Kits (Illumina Inc. San Diego, CA, USA). The average fragment size was determined using the 2100 Bioanalyzer instrument (Agilent Technologies). Cluster formation on the flow cell was performed using the cBot instrument (Illumina Inc.,). All libraries were sequenced on a HiSeq 2500 Version 4 (Illumina Inc.) running HCS software v2.2.6.

After sequencing, the Galaxy Project [30] interface to cloud computing resources was used to bring command-line-driven tools. The Illumina output files were converted to FASTQ file format and sequence quality trimming was performed using Trimmomatic [31], with a phred quality score > 30 over the length of the reads. The reads were aligned to the UMD3.1 *Bos taurus* 8 reference genome using Tophat v2.0.11 [32] and then assembled into transcripts using Cufflinks v2.2.1 [33] with genome annotation using UCSC Main Cow RefSeq genome. Reads per kilobase per million mapped reads (RPKM) were used to establish the transcript expression rate. Identification of the differentially expressed genes was performed using Cuffdiff v 2.2.1 software, and visualization was generated by CummeRbund [33]. Because a small number of genes may be highly expressed in the epithelial tissue and could hamper accurate quantification of genes with low expression, differential gene expression was defined as significant according to a false discovery rate (FDR) < 0.05, adjusted with the procedure of Benjamini and Hochberg [34].

### Functional annotation and pathway analyses of differentially expressed genes

For the functional annotation of differentially expressed genes, the Gene Ontology (GO) and UNIPROT databases were used to obtain functional information. The genes were searched for functions associated with the progression of papillomatous lesions in cattle, and the metabolic pathways in which the products of these genes participate were mapped with the KEGG database (Additional file 4). Protein-protein interactions was analyzed using the STRING database.

### qPCR verification of RNA sequencing results

The expression levels of nine differentially expressed genes (BOLA-DYB, DAPL1, KRT78, KRTAP3–1, UBA7, BNIP3, IVL, FABP4, and CST6) were analyzed using real-time quantitative PCR (qPCR) to validate RNA sequencing results. These genes were chosen because they present different functions important for the virus infection cycle. Two reference genes (RPS15 and GAPDH) were used following a published protocol [16]. Reverse transcription was performed with the High-Capacity cDNA Reverse Transcription kit (Thermo Fisher Scientific), using aliquots (2 μg) of the same total RNA used in RNA sequencing analysis. The cDNA samples were diluted to 20 ng/μl based on the starting concentration of RNA. The PCR reaction mix was composed of 12.5 μL Power SYBR® Green PCR Master Mix (Life Technologies Inc., Burlington, ON, Canada), 2.5 μL cDNA, 600 nM of each forward and reverse primers (Additional file 5). The thermal cycling conditions were 95 °C for 10 min, followed by 40 cycles at 95 °C for 15 s and 60 °C for 1 min. A melting curve was generated by the end of each reaction to verify the formation of a single peak and to exclude the possibility of non-specific product formation. The experiments were carried out in triplicate for each data point. Relative quantification of gene expression was determined using the 2^-ΔΔCt^ method [35]. A Student’s *t*-test was used to identify significant differences in gene expression (*p* < 0.05 was considered significant).

Primer validation was performed by PCR using the Master Mix PCR kit (Promega). For each reaction, 12 μL of PCR Master Mix, 1 μL of each primer, 9 μL of ultrapure water, and 2 μL of DNA was used. The PCR cycling conditions were as follows: initial denaturation at 94 °C for 10 min, followed by 35 cycles of 94 °C for 1 min, 60 °C for 1 min, and 72 °C for 1 min, and a final extension at 72 °C for 10 min. PCR products were visualized using 1.5% agarose gel electrophoresis.

## Additional files

Additional file 1: Reads and mapping statistics. (XLSX 11 kb)

Additional file 2: RPKM density histogram of transcripts from RNA-seq of BPV infected and non-infected groups. The diagram shows the distribution of the density of expressed genes at different RPKM levels. 2a) distribution of the density of expressed genes in infected animals. 2b) distribution of the density of expressed genes in non-infected animals. (PDF 96 kb)

Additional file 3: Differentially expressed genes between infected and healthy cows. (XLSX 80 kb)

Additional file 4: Functional annotation of differentially expressed genes. (XLSX 293 kb)

Additional file 5: Genes and primer sequences used in qPCR validation of RNA-sequencing data. (XLSX 12 kb)
