# Supplementary figures and images for: Comparative transcriptomic analysis of bovine papillomatosis
Source: BMC Genomics. 2018 Dec 19;19:949. doi: 10.1186/s12864-018-5361-y (PMC6300001; doi:10.1186/s12864-018-5361-y)

2a

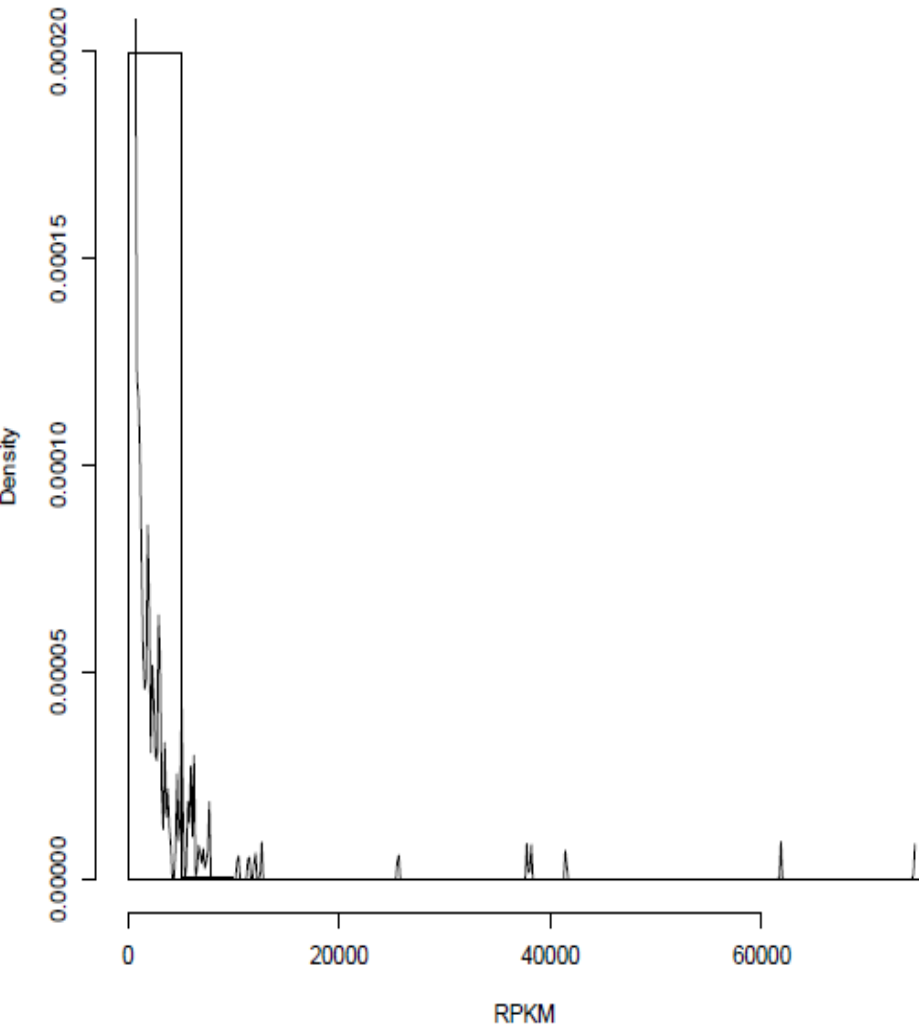

2b

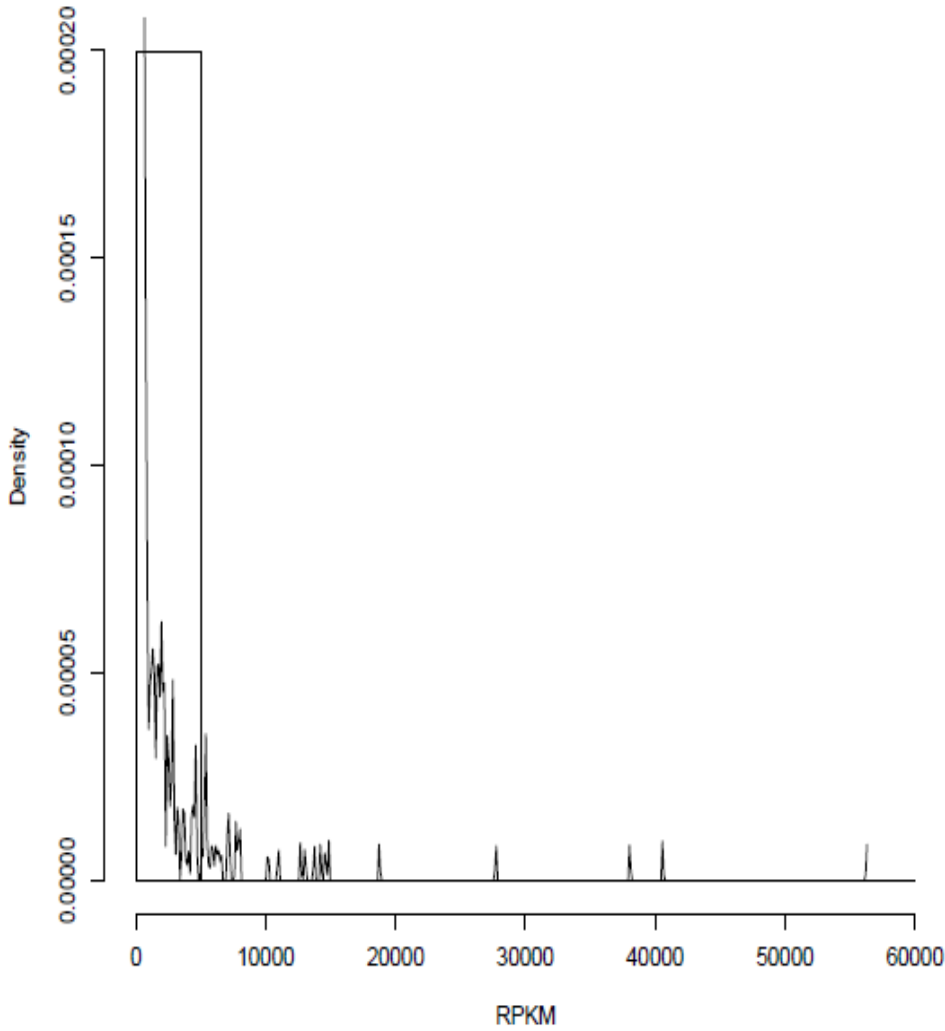

Supplement: Supplementary file 2 — RPKM density histogram of transcripts from RNA-seq of BPV infected and non-infected groups. The diagram shows the distribution of the density of expressed genes at different RPKM levels. 2a) distribution of the density of expressed genes in infected animals. 2b) distribution of the density of expressed genes in non-infected animals. (PDF 96 kb) [file 12864_2018_5361_MOESM2_ESM.pdf]
